# Influence of the Culture Substrate on the *Agaricus* *blazei* Murrill Mushrooms Vitamins Content

**DOI:** 10.3390/plants8090316

**Published:** 2019-08-30

**Authors:** Sándor Rózsa, Dănuț-Nicolae Măniuțiu, Gheorghe Poșta, Tincuța-Marta Gocan, Ileana Andreica, Ileana Bogdan, Melinda Rózsa, Vasile Lazăr

**Affiliations:** 1 Horticulture and Landscape, Faculty of Horticulture, University of Agricultural Sciences and Veterinary Medicine Cluj-Napoca, 400372 Cluj-Napoca, Romania; 2 Horticulture, Faculty of Horticulture, Banat University of Agricultural Sciences and Veterinary Medicine “King Mihai I of Romania”, 300645 Timisoara, Romania; 3 Economics, Faculty of Horticulture, University of Agricultural Sciences and Veterinary Medicine Cluj-Napoca, 400372 Cluj-Napoca, Romania; 4 Technical Sciences and Soil Sciences, Faculty of Agriculture, University of Agricultural Sciences and Veterinary Medicine Cluj-Napoca, 400372 Cluj-Napoca, Romania

**Keywords:** *Agaricus blazei* Murrill, culture substrate, protein addition, vitamin content

## Abstract

The vitamin content of cultivated mushrooms differs from one species to another, depending on their stage of development, the nutrient substrate used to produce them, and the microclimate in the culture space. *Agaricus blazei* Murrill is one of the most popular cultivated medicinal mushrooms, with scientifically proven therapeutic properties. Considering that the *Agaricus* spp. mushrooms culture substrate can be produced using various raw materials, in this paper we have studied the influence of the culture substrate using four types of substrate with different protein additions on the vitamin content of mushrooms. The food qualities of the *Agaricus* *blazei* Murrill mushrooms, evaluated by the chemical composition, generally revealed the product obtained on the classic compost, improved with the addition of proteinaceous of corn flour. Mushrooms harvested on this substrate have the highest levels of B_1_ (1151 μg 100 g^−1^ dm), B_9_ (671 μg 100 g^−1^ dm), B_12_ (906 μg 100 g^−1^ dm), PP (55.33 μg 100 g^−1^ dm), and C vitamins (21.67 μg 100 g^−1^ dm). The content of ergosterol, as a precursor of D_2_ vitamin, has higher values in the product obtained on the classic compost, with the addition of wheat bran (90.17 mg 100 g^−1^ dm) and the addition of corn flour (94 mg 100 g^−1^ dm).

## 1. Introduction

The food value of a product depends mainly on two factors, as follows: The digestibility of the compounds that make up its composition and the chemical composition [1]. The chemical analyses of common edible mushrooms showed that, on the basis of fresh substance, the moisture content is 89–91%, the ash is 0.97–1.26%, the protein is 1.78–4.94%, the fat is 0.1–0.65%, crude fiber is 0.09–1.67%, carbohydrates are 2.3–6.28%, and the energy value is 24.4–34.4 Kcal. Glucose is present in very small amounts and, like any other vegetable, the mushrooms contain very small amounts of fat. Their caloric value is rather low compared to other foods, so they can be recommended in slimming diets [2,3].

The vitamin content shows that among all plant products, mushrooms are real sources of vitamins, especially those of complex B (thiamine, biotin, nicotinic acid, pantothenic acid) and vitamin D, which is no longer found in any other plant, being specific to fish lard and meat [4]. In addition, in cultivated mushrooms there are significant amounts of vitamin A, A_1_, and vitamin C (ascorbic acid). With regard to vitamin C in mushrooms, it has been determined that under diffuse light conditions, its content increases and, in the absence of light, decreases. The mushrooms also contain significant amounts of organic acids, predominantly fumaric, succinic, malic, and less citric acid [5].

The use of therapeutic mushrooms is one of the most exciting areas of natural medicine, providing a significant therapeutic benefit, backed by a long history of traditional use and more and more scientific evidence [6].

It is hypothesized that cultured mushrooms, especially *Agaricus* spp., are potential stimulants of metabolic actions that are capable of stopping and curing various cancers [7].

At the international level, research into the production of nutraceutical supplements obtained from the biomass of various species of edible and medicinal basidiomycetes is quite advanced. In-depth studies carried out in China, Japan, the USA, and Russia have demonstrated the beneficial effects of producing dietary supplements from the fungal nutrient biomass obtained by cultivating edible basidiomycetes of the species *Cordyceps sinensis*, *Agaricus blazei*, *Grifola frondosa*, *Ganoderma lucidum*, *Lentinula edodes*, *Pleurotus ostreatus*, *Schizophyllum commune*, and *Trametes versicolor*, for use in the prevention and treatment of multiple human disorders [8].

Although it is one of the mushrooms with therapeutic effects, *Agaricus blazei* Murrill has quickly become one of the most popular cultivated medicinal mushrooms. It has been reported in a study as being consumed by 31% of urogenital cancer patients in Japan [9], has the fastest growing US sales of any fungus [10,11,12], and is one of the three most popular medicinal mushrooms in Taiwan. The white-mushroom-related species, *Agaricus bisporus*, has a broad clinical setting [11,12,13].

*Agaricus blazei* Murrill is a mushroom originating from Brazil, known as the “Sun Mushroom”. Currently, in several Eastern countries, this mushroom is consumed both as an edible fungus and for therapeutic purposes, especially in the prevention and treatment of cancer [14,15]. Several studies reviewed by Wisitrassameewong et al. [16] highlight the importance of *Agaricus blazei* Murrill’s medicinal properties. It has been traditionally used to treat many common diseases, such as atherosclerosis, hepatitis, hyperlipidemia, diabetes, dermatitis, and cancer [14,15,16,17,18].

All edible mushrooms are rich in B vitamins plus other vitamins such, as vitamin C and ergosterol. The mushrooms have historically been used as medicines and tonics [19]. Studies have shown that fruit organisms also contain minerals and other vitamins, especially B_1_, B_2_, and niacin [20,21,22,23].

Bioactive compounds in fungi can be isolated from fruit plants or by extraction from pure mycelial culture [24]. It has been reported that the *Agaricus blazei* Murrill mushroom produces various bioactive compounds that have the potential to treat several diseases [13]. This mushroom has been used as a food for the prevention of cancer, diabetes, hyperlipidemia, arteriosclerosis, and chronic hepatitis and is known as a stimulator of the immune system [25].

Apart from the fact that the mushrooms are a food and even therapeutic, or because of that, the mushroom culture also has some economic advantages. Through preservation, the mushrooms do not change organoleptic parameters and dehydration considerably prolongs the shelf life. [26].

Mushrooms provide important sources of protein extracted from materials of very low economic value, such as manure, agricultural waste, forestry, and the wood industry. Using the maximum yield of the nutrient substrate, which can be reused as a crop fertilizer for agriculture (*Agaricus*, *Coprinus*, *Stropharia* mushrooms), as a feed for animals, or as fuel (*Pleurotus* mushrooms) after a 3–5 month crop cycle [27].

The primary components of the culture medium of the *Agaricus* genus mushrooms are the most commonly locally available composting materials, e.g., agricultural waste rich in lignocellulosic complexes, straw, cotton sponges, herbs, sawdust enriched with manure, poultry manure, wheat bran, rice bran, and calcium [19,28,29,30,31,32].

Wang et al. [33] established the applicability of post-harvest asparagus residues in the cultivation of *Agaricus blazei* Murrill. In turn, Gern et al. [34] successfully used the used substrate remaining after the cultivation of *Pleurotus* spp., with the addition of rice bran. The substrate is fermented during composting, with microbiological changes and changes in the C:N ratio occurring during the composting process [35]. As reported by the same author, during fermentation, typically 23–25 days, the temperature should not exceed 60 °C. A study by Gonzalez et al. [36] showed that uncompacted substrate can also be used in the cultivation of *Agaricus blazei* Murrill.

Increasing the nutritional quality of mushroom compost is a prime factor in increasing yield. Schisler and Sinden [37] have shown that when the compost was supplemented with different seeds ground together with refined and crude seed oils applied to the compost before applying the coating, the production of mushrooms increased. An increase in the number and yield of mushrooms was also reported when some chemicals were sprayed as a nutritional supplement on compost [38].

Protein rich supplements, such as soy beans (*Glycine max* L.), beans (*Phaseolus vulgaris* L.), and peas (*Pisum sativum* L.) added to compost significantly stimulated mushroom production, shortened the cycle time of the culture, and have rushed to the substrate, compared with blank samples without supplements. The biological efficacy (BE) ranged from 26.1% in the unsupported substrate to 73.1% in the compost supplemented with soybean (*Glycine max* L.). No significant differences in the yield of mushrooms observed between the evaluated supplements were observed [39].

The nutrient substrate must present degradation or microbial synthesis products in an assimilable form as quickly as possible by the mushroom, having a biochemical specificity corresponding to the metabolic requirements of the mushroom [40].

The raw material for preparing the nutrient substrate for mushroom culture requires various basic components and support components, organo-mineral amendments, and fertilizers [41].

The basic component is manure and the support components are as follows: Straw, hay, corn, sawdust, shavings. Amendments with calcium, calcium sulfate and calcium carbonate, respectively, are administered at a rate of 16–25 kg per ton. Calcium is indispensable for mycelium growth and mushroom formation. When preparing compost, calcium sulfate is used more frequently than calcium carbonate [42].

Mineral substances used to prepare the nutrient substrate provide the source of nitrogen (technical urea) and calcium. Calcium is the indispensable element for mycelium because its presence eliminates the inhibitory effect of other elements [43].

Considering the medical importance of the nutraceutical supplements obtained from *Agaricus blazei* Murrill mushrooms and the fact that many mushroom producers use the commercial second phase substrate produced for the *Agaricus bisporus* mushrooms cultivation to grow *Agaricus blazei* Murrill mushrooms, considering that this mushroom is of high importance in the prevention and treatment of different diseases, we considered it necessary to adapt the standard recipes, used in industrial *Agaricus bisporus* mushrooms, by tracking and directing the physicochemical factors during the composting period [4] and supplementing the obtained substrate with different protein additions that are easy to obtain, depending on what we want the final product to contain.

At the same time, using a composting facility [4], where the physical and chemical factors of the substrate are monitored and directed, different combinations of substrates can be tested, obtaining new substrate recipes, which subsequently, if the final product obtained from it meets our requirements, ensure a balance between the bioactive substance content. It can also be used on an industrial scale.

Given the fact that many industries are currently generating a lot of lignocellulosic waste, which could be used in the mushroom industry as a culture substrate or as additives in different substrate recipes, we hope this study will be useful for testing other species of mushrooms or substrate recipes, thus adding value to agricultural operations and waste reduction.

## 2. Results

The content of *Agaricus blazei* Murrill mushrooms in bioactive substances, related to the dry matter obtained on the four types of substrate with various protein additions used in the experiment, is presented in the Table 1.

### 2.1. Ergosterol Content of Agaricus blazei Murrill Mushrooms (mg 100 g^−1^ dm)

**Figure 1:**
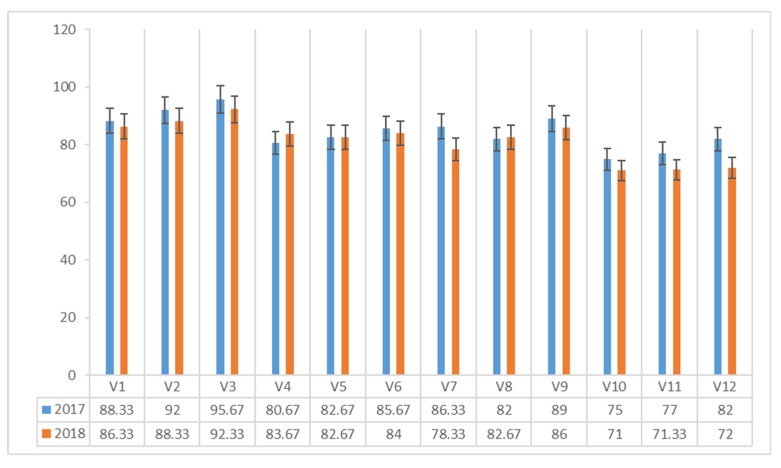
Ergosterol content of *Agaricus blazei* Murrill mushrooms (mg 100 g^−1^ dm) in two years of experiments.

The content of ergosterol (mg 100 g^−1^ dm) in *Agaricus blazei* Murrill mushrooms varied between 75.00 and 95.67 mg 100 g^−1^ dm in 2017 and 71.00 and 92.33 mg 100 g^−1^ dm in 2018 (Figure 1).

In both experimental years, the maximum value was recorded in the mushrooms harvested on the classical compost with a protein admixture of corn flour (V3), with a maximum of 95.67 mg 100 g^−1^ dm in 2017 and 92.33 mg 100 g^−1^ dm in 2018.

The lowest content of ergosterol was recorded in mushrooms harvested from cane compost without additional protein supplement (V10), with 75.00 mg 100 g^−1^ dm in 2017 and 71.00 mg 100 g^−1^ dm in 2018.

### 2.2. Vitamin C Content of Agaricus blazei Murrill Mushrooms (mg 100 g^−1^ dm)

**Figure 2:**
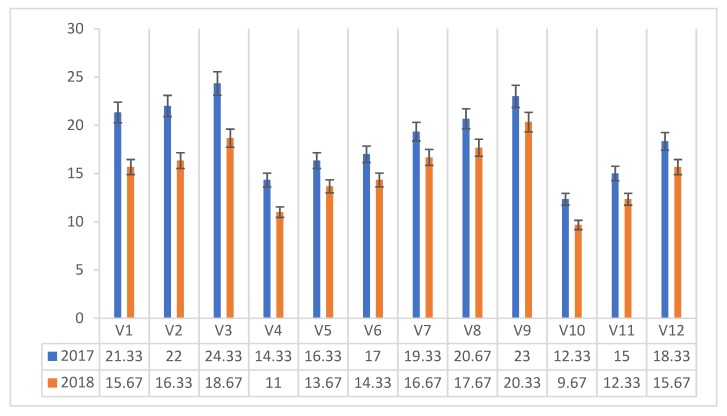
Vitamin C content of *Agaricus blazei* Murrill mushrooms (mg 100 g^−1^ dm) in two years of experiments.

The vitamin C content (mg 100 g^−1^ dm) in *Agaricus blazei* Murrill mushrooms varied between 12.33 and 24.33 mg 100 g^−1^ dm in 2017 and 9.67 and 20.33 mg in 2018 (Figure 2).

In 2017, the maximum value was recorded for the mushrooms harvested on the classical compost with added corn flour protein (V3), with a maximum of 24.33 mg 100 g^−1^ dm, in 2018, the maximum value was recorded for mushrooms harvested from the composite compost with additional corn flour protein addition (V9).

Following the effect of interaction, protein × compost, in the years of experimentation on the vitamin C content (mg 100 g^−1^ dm) in *Agaricus blazei* Murrill mushrooms, it can be seen that the highest vitamin C content was recorded in the mushrooms harvested on the composite compost with the corn flour protein admixture (V9), with 21.67 mg 100 g^−1^ dm, followed by the mushrooms harvested from the classic compost with corn flour (V3) with 21.50 mg 100 g^−1^ dm of vitamin C.

### 2.3. B_1_ Vitamin (Thiamin) Content of Agaricus blazei Murrill Mushrooms (μg 100 g^−1^ dm)

**Figure 3:**
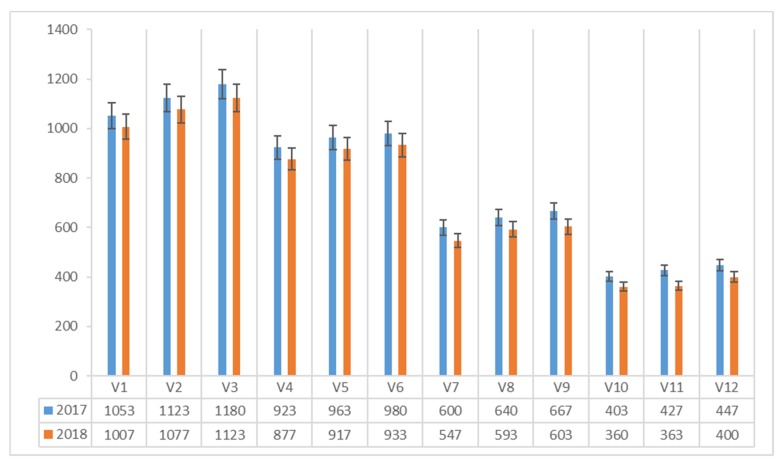
Vitamin B_1_ content of *Agaricus blazei* Murrill mushrooms (μg 100 g^−1^ dm) in two years of experiments.

The content of vitamin B_1_ (μg 100 g^−1^ dm) in *Agaricus blazei* Murrill mushrooms, ranged between 403 and 1180 μg 100 g^−1^ dm in 2017 and between 360 and 1123 μg 100 g^−1^ dm in 2018 (Figure 3). In both experimental years, the maximum value was recorded in the mushrooms harvested on the classical compost with the corn flour protein admixture (V3).

Analyzing the effect of the compost × additive interaction, on average, over the years of the experimental trial on the vitamin B_1_ content (μg 100 g^−1^ dm), it can be noticed that the protein addition with corn flour (A3) increased the vitamin B_1_ content of *Agaricus blazei* Murrill mushrooms on all four types of compost.

The highest values were obtained on the classical (C1) (1151 μg 100 g^−1^ dm) and the synthetic compost (C2) (956 μg 100 g^−1^ dm).

### 2.4. Vitamin B_2_ (Thiamin) Content of Agaricus blazei Murrill Mushrooms (μg 100 g^−1^ dm)

**Figure 4:**
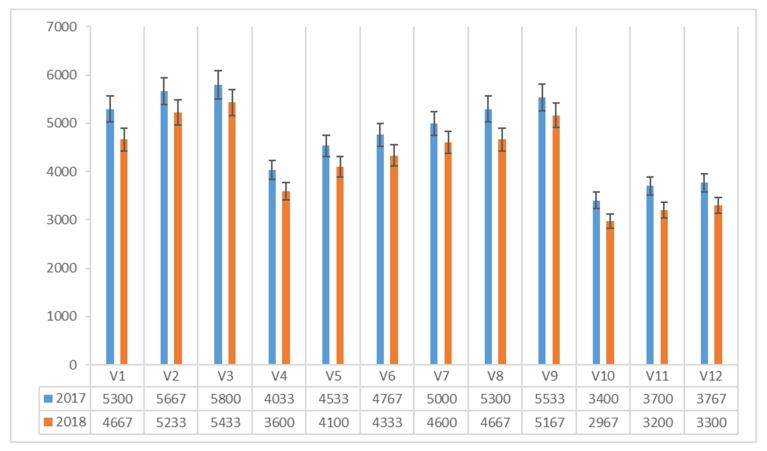
Vitamin B_2_ vitamin content of *Agaricus blazei* Murrill mushrooms (μg 100 g^−1^ dm) in two years of experiments.

The content of vitamin B_2_ (μg 100 g^−1^ dm) in *Agaricus blazei* Murrill mushrooms ranged between 3400 and 5800 μg 100 g^−1^ dm in 2017 and between 2967 and 5433 μg 100 g^−1^ dm in 2018 (Figure 4). In both experimental years, the maximum value was recorded in the mushrooms harvested on the classical compost with the corn flour protein admixture (V3).

Analyzing the effect of the compost × additive interaction in years of experimental trial on the vitamin B_2_ content (μg 100 g^−1^ dm) of *Agaricus blazei* Murrill, it can be noticed that the protein addition of corn flour (A3) influenced the vitamin B_2_ content of the mushrooms, the first place being the combination of corn flour protein admixture with classical compost (V3), with 5616 μg vitamin B_2_ 100 g^−1^ dm. The composite compost with wheat bran protein (V8), also influenced the vitamin B_2_ content of the *Agaricus blazei* Murrill mushrooms (4983 μg 100 g^−1^ dm), but with statistically uninsured differences compared to the first variation.

### 2.5. Vitamin B_9_ (Folic Acid) Content of Agaricus blazei Murrill Mushrooms (μg 100 g^−1^ dm)

**Figure 5:**
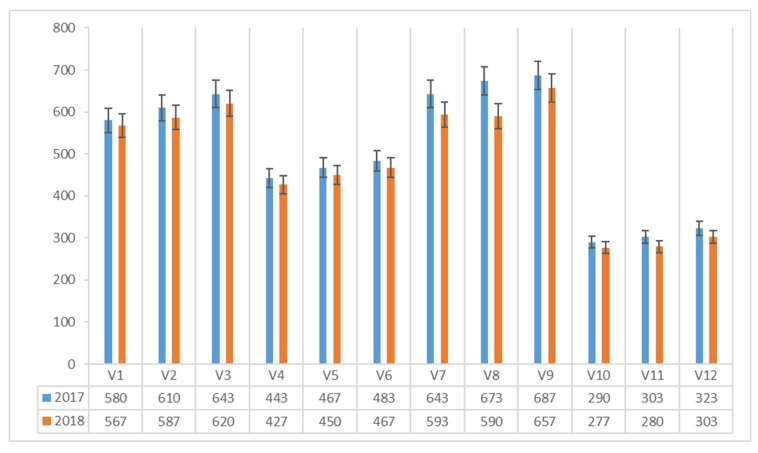
Vitamin B_9_ content of *Agaricus blazei* Murrill mushrooms (μg 100 g^−1^ dm) in two years of experiments.

The vitamin B_9_ content (μg 100 g^−1^ dm) in *Agaricus blazei* Murrill mushrooms ranged between 290 and 687 μg 100 g^−1^ dm in 2017 and between 277 and 657 μg 100 g^−1^ dm in 2018 (Figure 5).

The mushrooms harvested from the classic and mixed composts with the added corn flour addition (V3, V9), achieved the highest values of vitamin B_9_ content.

### 2.6. Vitamin B_12_ (Cobalamin) Content of Agaricus blazei Murrill Mushrooms (μg 100 g^−1^ dm)

**Figure 6:**
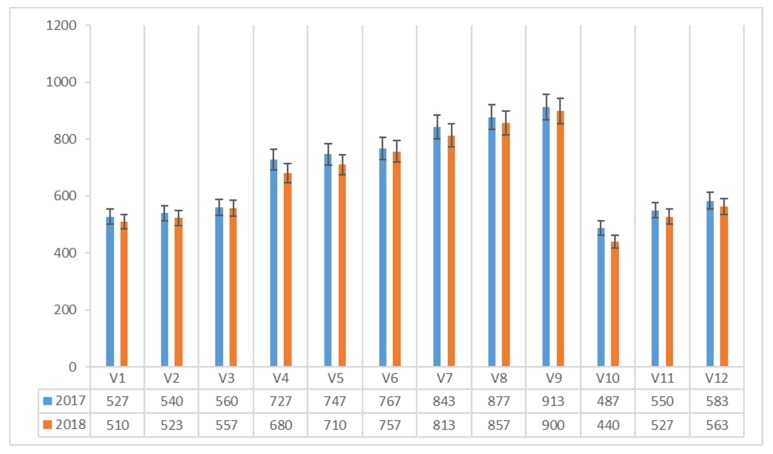
Vitamin B_12_ content of *Agaricus blazei* Murrill mushrooms (μg 100 g^−1^ dm) in two years of experiments.

The content of vitamin B_12_ (μg 100 g^−1^ dm) in *Agaricus blazei* Murrill mushrooms, ranged between 487 and 913 μg 100 g^−1^ dm in 2017 and between 440 and 900 μg 100 g^−1^ dm in 2018 (Figure 6). In both experimental years the maximum value was recorded in the mushrooms harvested on the composite compost with the proteinaceous addition of corn flour (V9).

The highest content of vitamin B_12_ was found in mushrooms grown on mixed compost, especially that with the additional protein supplementation of corn flour (V9), followed closely, but with statistically ensured differences, by protein addition of bran wheat (V8).

### 2.7. PP Vitamin (B_3—_niacin) Content of Agaricus blazei Murrill Mushrooms (mg 100 g^−1^ dm)

**Figure 7:**
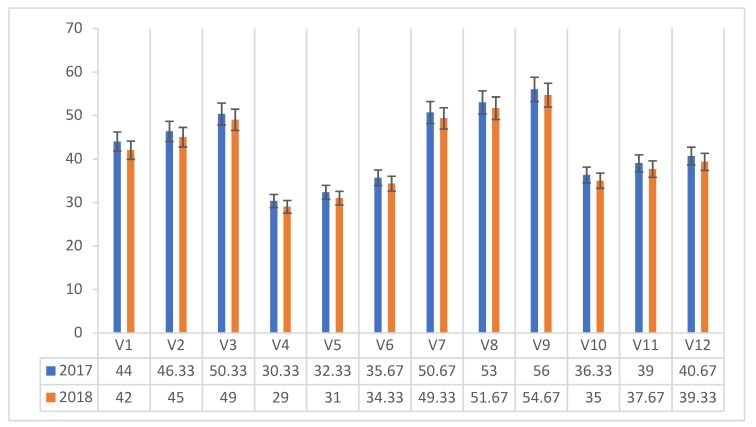
PP vitamin content of *Agaricus blazei* Murrill mushrooms (mg 100 g^−1^ dm) in two years of experimental trial.

The content of the PP vitamin (mg 100 g^−1^ dm) in *Agaricus blazei* Murrill mushrooms ranged between 30.33 and 56 mg 100 g^−1^ dm in 2017 and between 29 and 54.67 mg 100 g^−1^ dm in 2018, depending on the experimental compost variants used (Figure 7). In both experimental years the maximum value was recorded in the mushrooms harvested on the composite compost with the proteinaceous addition of corn flour (V9).

As with other B-complex vitamins, PP vitamin levels are higher in mushrooms grown on composite compost (C3), with or without protein addition. The highest value (55.33 mg 100 g^−1^ dm) was obtained with the use of the protein admixture of corn flour (A3).

## 3. Discussion

Compared with the *Agaricus bisporus* mushrooms, the ergosterol content of the *Agaricus blazei* Murrill mushroom is lower compared to the values presented by other researchers.

Thus, Teichmann et al. [44] mentioned 399–474 mg ergosterol 100 g^−1^ dm, Barreira et al. mentioned [45] 77–352 mg 100 g^−1^ dm, Villares et al. mentioned [46] 642 mg/100 g^−1^ dm, and Stojkovic et al. mentioned [18] 139 mg/100 g^−1^ dm, but the analyzed works did not specify the type of substrate from which the mushrooms were harvested.

Vitamin C, is related to the C6 sugars, being the aldono-1,4-lactone of a hexonic acid (L-galactonic or L-gulonic acid) and contains an endiolic group at 2 and 3 carbon atoms. Its synthesis may be influenced by the presence of proteins [47,48,49], in the case of our study, the vitamin C content is influenced by the protein addition used in the culture substrate.

Regarding the vitamin C content of the *Agaricus blazei* Murrill mushrooms, studies conducted by Tsai et al. [50] determined a content of 14.25 mg vitamin C 100 g^−1^ dm, Carneiro et al. determined a content of [51] 21.23 mg 100 g^−1^ dm, and Cohen et al. determined a content of [52] 16.42 mg 100 g^−1^ dm. The values determined by us were found within these ranges.

Compared with the *Agaricus bisporus* mushrooms, the vitamin C content of *Agaricus blazei* Murrill mushroom is similar.

Thus, Mattila et al. [53] mentioned a content of 19 mg vitamin C 100 g^−1^ dm, Furlani and Godoy [54] mentioned a content of 18.01 mg 100 g^−1^ dm, and Bernas and Jaworska mentioned a content of [55] 19.17 mg 100 g^−1^ dm.

Vitamin C sometimes reacts with vitamin E, producing a vitamin C radical and regenerating vitamin E. Both radicals are poorly reactive species because of their unpaired electron [56,57].

Comparing the vitamin B1 content of *Agaricus blazei* Murrill mushrooms with other foods that are known thiamine sources, such as cereals, this is lower, but is close to those contained in vegetables and higher than in eggs. [58].

Regarding the vitamin B_1_ content of *Agaricus blazei* Murrill mushroom, Tsai et al. [50] mention the amount of 720 μg 100 g^−1^ dm, Carneiro et al. [51] mention the amount of 940 μg 100 g^−1^ dm, and Cohen et al. [52] mention the amount of 1150 μg 100 g^−1^ dm. The values determined by us are found within these ranges.

Compared with the *Agaricus bisporus* mushrooms, the vitamin B1 content of the *Agaricus blazei* Murrill mushroom obtained by us is higher and is confirmed by other authors.

Thus, Mattila et al. [53] mentions 920 μg 100 g^−1^ dm, Furlani and Godoy [54,58] mention 630 μg 100 g^−1^ dm, and Bernas and Jaworska [55] mention 710 μg 100 g^−1^ dm.

Regarding the B_2_ vitamin content of the *Agaricus blazei* Murrill mushrooms, Tsai et al. [50] found the amount to be 3550 μg 100 g^−1^ dm, Carneiro et al. [51] found the amount to be 4625 μg 100 g^−1^ dm, and Cohen et al. [52] found the amount to be 6200 μg 100 g^−1^ dm. The values determined by us are found within these ranges.

Compared with the *Agaricus bisporus* mushrooms, the B_2_ vitamin content of the *Agaricus blazei* Murrill mushroom has lower values. Thus, Mattila et al. [53] mention 5100 μg 100 g^−1^ dm, Furlani and Godoy [54] mention 2500 μg 100 g^−1^ dm, and Bernas and Jaworska [55] mention 1800 μg 100 g^−1^ dm

Since the analyzed samples were from different growing substrates, different protein addition could have contributed to these differences. As mentioned by other authors, the different substrates used for cultivation could also have altered the composition of the mushrooms [59,60].

Regarding the vitamin B_9_ content of *Agaricus blazei* Murrill mushroom, Cohen et al. [52] determined the value of 220 μg 100 g^−1^ dm, so the values determined by us exceed those found by other authors. In this case, the results obtained were influenced by the protein addition from the culture substrate. In the literature, we have not found data on the vitamin B_9_ content in mushrooms grown on different substrates.

Compared with *Agaricus bisporus*, the B_9_ vitamin content of *Agaricus blazei* Murrill mushroom is almost identical to that presented by Mattila et al. [53], 430 μg 100 g^−1^ dm, by Furlani and Godoy [54], 240 μg 100 g^−1^ dm, and by Bernas and Jaworska [55], 750 μg 100 g^−1^ dm.

Detection of vitamin B_9_ (Folic acid) in all experimental variants, within the range of 290 to 687 μg 100 g^−1^ dm in 2017 and from 277 to 657 μg 100 g^−1^ dm in 2018, shows that these mushrooms are rich in vitamin B9. *Agaricus blazei* Murrill mushrooms are a rich source of protein, vitamins, and minerals [61,62], showing a high interest as a source of raw material in the pharmaceutical industry, with folic acid being successfully used in the treatment of anemia and for pregnant women [63].

Analysis of vitamin B_12_ in harvested mushrooms varied from variant to variant, with higher concentrations of B_12_ detected in the outer peel than in the cap, stalk, or flesh, suggesting that vitamin B_12_ is probably bacteria-derived [64]. Higher concentrations of vitamin B_12_ were also detected in the mushrooms harvested from mixed substrate with protein addition (C3 A2, C3 A3). HPLC and mass spectrometry showed that vitamin B_12_ retention time and mass spectra were identical to those of the standard B_12_ vitamin and those of food products.

Regarding the vitamin B_12_ content of the *Agaricus blazei* mushroom, they are found within the ranges found in the literature of Carneiro et al. [51], 920 μg 100 g^−1^ dm, and Cohen et al. [52], 760 μg 100 g^−1^ dm.

PP vitamin (B_3_-Niacin), contained in mushrooms, helps the human body to produce energy from blood sugar, helps to reduce the cholesterol levels, gives the skin a healthy and shiny texture, and stimulates the production of progesterone, estrogen, and testosterone. [65].

Regarding the PP vitamin content of the *Agaricus blazei* Murrill fungus, Tsai et al. [50] found the amount to be 45 mg 100 g^−1^ dm, Carneiro et al. [51] found the amount to be 53 mg 100 g^−1^ dm, and Cohen et al. [52] found the amount to be 36 mg 100 g^−1^ dm. The values we determined are found within these ranges.

Compared with the *Agaricus bisporus* mushrooms, the PP vitamin content of *Agaricus blazei* Murrill mushroom is similar. This is also asserted by Mattila et al. [53], 54 mg 100 g^−1^ dm, Furlani and Godoy [54], 39 mg 100 g^−1^ dm, and Bernas and Jaworska [55], 46 mg 100 g^−1^ dm.

Following the results presented above, it can be said that the consumption of mushrooms can increase the intake of vitamins in certain diets.

Following the evolution of the market for food supplements, especially those obtained from mushrooms with a scientifically proven therapeutic effect, it can be said that the demand for such food supplements is increasing. If it is desired to increase the content of certain vitamins in these food supplements, one can intervene on the substrate recipes, thus influencing the chemical composition and the content in bioactive substances of the finished product. In addition, if the products obtained from a specific recipe have a high level of bioactive substances, but also the level of production has a high biological efficiency, then that recipe can also be made at the industrial level, without causing significant losses, with the initial tests being carried out on small amounts of substrate.

## 4. Materials and Methods

### 4.1. Biological Material Used in Experiments

**Figure 8:**
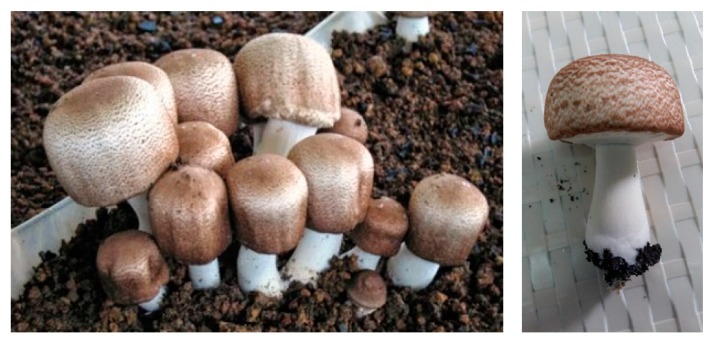
*Agaricus blazei* Murrill mushrooms. Source: Photo original—SC CIUPERCĂRIA SRL, Aghireșu-Fabrici, Cluj County.

The biological material that has been used in the experimental trial comes from a pure culture and is represented by the M7700 strain. The species *Agaricus blazei* Murrill (Figure 8), is popularly called “the royal fungus”, “God’s mushroom”, “the goddess’s mushroom”, and “the fungus of the sun”.

**Figure 9:**
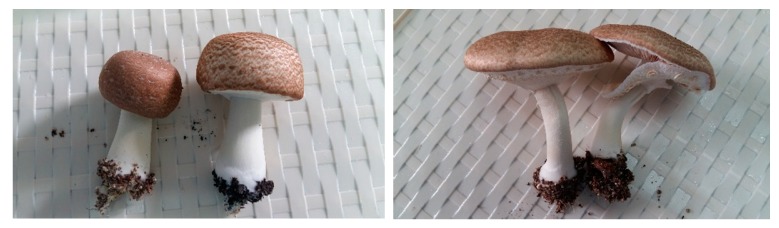
*Agaricus blazei* Murrill mushrooms hat. Source: Photo original—SC CIUPERCĂRIA SRL, Aghireșu-Fabrici, Cluj County.

The cap is thick, fleshy, hard, small to large in size, 5–11 cm in diameter, semi-globular at the beginning semi-globular then convex, smooth on the edges with pores in the center, white, yellow-creamy like an almond, or light brown to dark brown. On the edge of the hat are stuck pieces of veil. It has an almond flavor (Figure 9).

**Figure 10:**
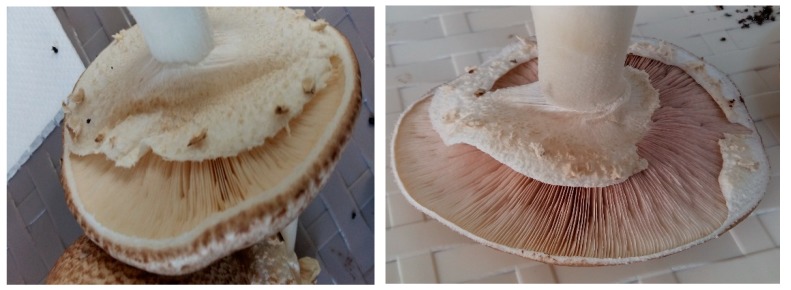
*Agaricus blazei* Murrill mushrooms blades. Source: Photo original—SC CIUPERCĂRIA SRL, Aghireșu-Fabrici, Cluj County.

The lamellas are free, dense, 8–10 mm wide, and have a white/pale pink color when young and brown-chocolate later (Figure 10).

Basidiospores are dark brown, up to 6–8 × 4–5 microns, have a chocolate color, are wide-elliptical to oval, and have no epispore.

**Figure 11:**
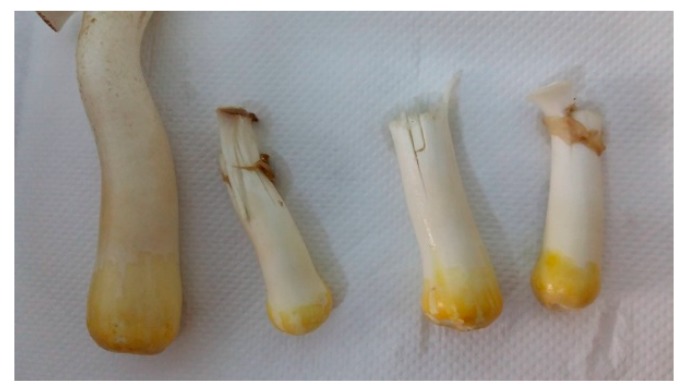
Yellowing the *Agaricus blazei* Murrill mushroom foot after removing the mycelian base. Source: Photo original—SC CIUPERCĂRIA SRL, Aghireșu-Fabrici, Cluj County.

The stipe is short and thick, like a column, filled in, cylindrical, has a white color, and is attached to a mycelian base. On the touch by hand, the leg of the mushroom becomes yellow (Figure 11). A ring remains of the leg after the velvet breaks. The length is 6–13 cm and the diameter is 1–3 cm.

### 4.2. Experimental Factors

To study the influence of compost recipes on the quality of mushroom production, as well as on the vitamin content of mushrooms, a bifactorial experimental trial was designed.

Factor A was the culture substrate with 4 graduations, as follows: C1—classic, C2—synthetic, C3—mixed, and C4—reed + horse manure. Factor B was the protein addition with 3 graduations, as follows: A1—without added protein supplement, A2—3% wheat bran, and A3—3% flour.

The combination of experimental factors resulted in 12 variants, presented in Table A1.

The compost recipes used in the experiment and the preparation process thereof are those described by Rozsa et al. [66] (Table A2).

**Figure A1:**
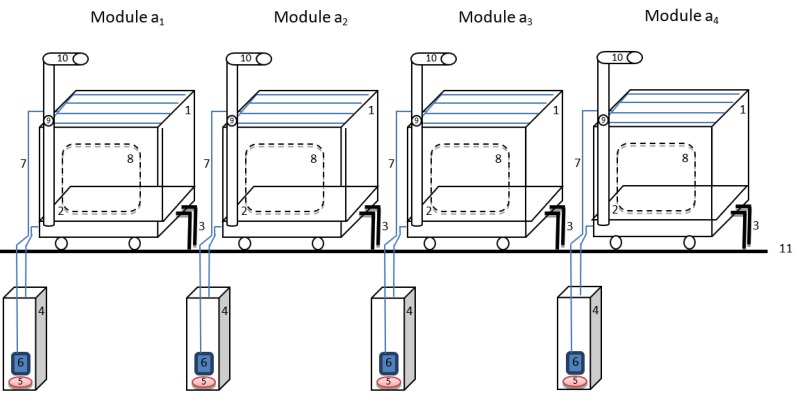
Sketch for the composting facility—4 modules [68]. 1—tank with capacity of 1 m^3^ for compost components; 2—rack for compost; 3—tank heating system for composting; 4—tank for the collection and recirculation the water excess, built underground; 5—heating elements for wetting water (purine); 6—water/purine recirculation pump; 7—recirculation pipes for wetting water/purine; 8—compost discharge door; 9—air flow control valve for aerobic composting; 10—air inlet pipe to aerobic composting from the compressor; 11—ground level.

**Figure A2:**
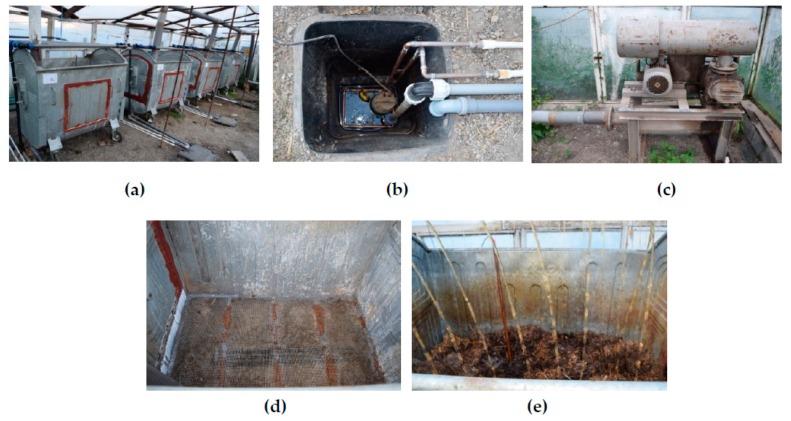
Sketch for the composting facility: (**a**) Composting facility—4 modules; (**b**) tank for the collection and recirculation of the water excess, built underground; (**c**) compressor for the air flow; (**d**) tank for composting, internal view; (**e**) compost components pre-soaking [68]. Source: Photo original—SC CIUPERCĂRIA SRL, Aghireșu-Fabrici, Cluj County.

Considering that in the experiment factor A—the culture substrate, had 4 graduations, for the directed composting, a 4-composting box was designed to control, realize, and record the optimum environmental conditions for composting (Figure A1, Figure A2a–e).

**Figure A3:**
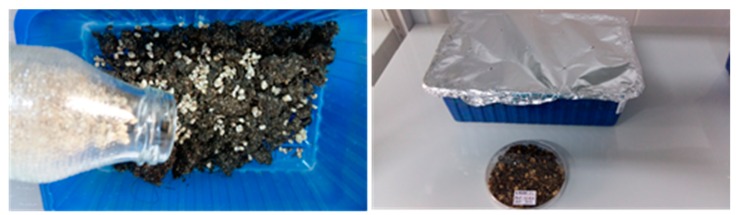
Compost sowing with *Agaricus Blazei* Murrill mushroom mycelium: (**left**) compost sowing; (**right**) sow tray covered with aluminum foil. Source: Photo original—SC CIUPERCĂRIA SRL, Aghireșu-Fabrici, Cluj County.

**Figure A4:**
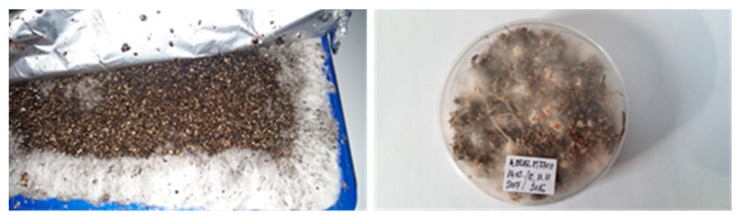
*Agaricus blazei* Murrill mushroom mycelium studding: (**left**) studded compost; (**right**) control Petri dishes with studded compost. Source: Photo original—SC CIUPERCĂRIA SRL, Aghireșu-Fabrici, Cluj County.

**Figure A5:**
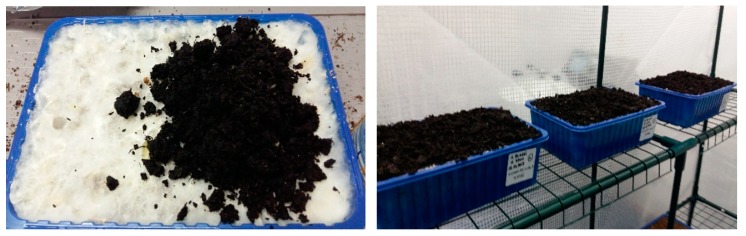
(**left**) Covering the mycelium coated substrate; (**right**) covered compost trays. Source: Photo original—SC CIUPERCĂRIA SRL, Aghireșu-Fabrici, Cluj County.

**Figure A6:**
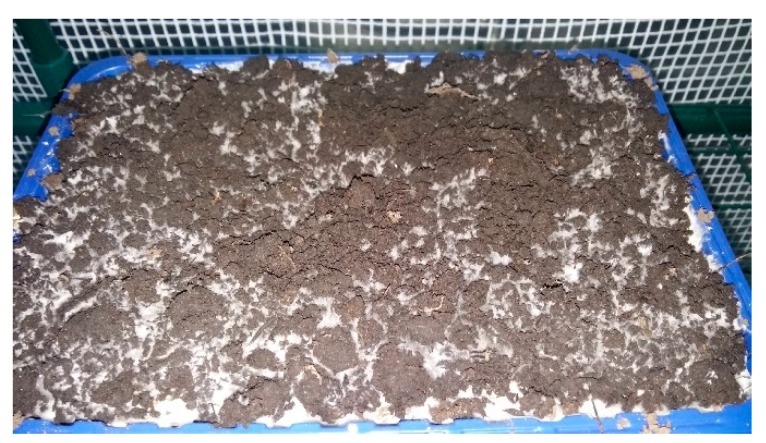
Coated cover layer with mycelium. Source: Photo original—SC CIUPERCĂRIA SRL, Aghireșu-Fabrici, Cluj County.

**Figure A7:**
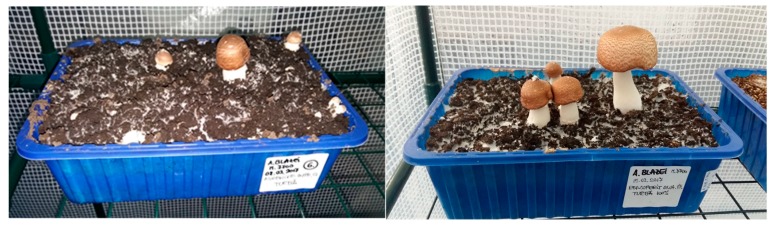
Formation of fructification primes: (**left**) V6 experimental variant; (**right**) V9 experimental variant. Source: Photo original—SC CIUPERCĂRIA SRL, Aghireșu-Fabrici, Cluj County.

**Figure A8:**
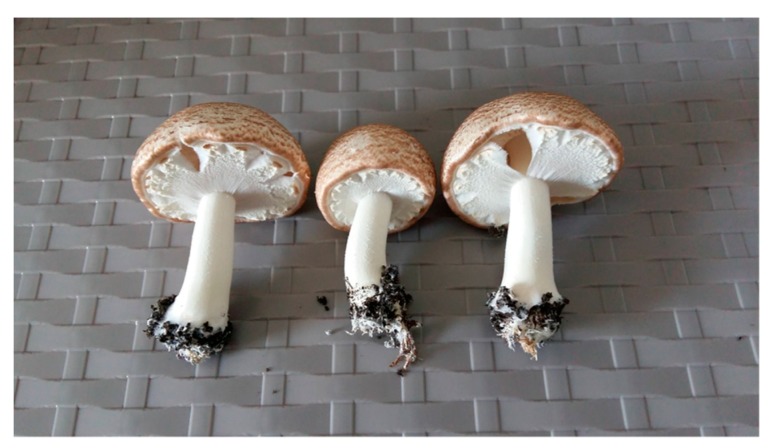
Harvested *Agaricus blazei* Murrill mushrooms. Source: Photo original—SC CIUPERCĂRIA SRL, Aghireșu-Fabrici, Cluj County.

The stages of mushroom culture are presented in the Appendix B, Figure A3, Figure A4, Figure A5, Figure A6, Figure A7 and Figure A8.

### 4.3. Standards and Reagents

Deionized distilled water (ddH_2_O) was prepared with a Favorit W4L water distillation system, ethanol (60%, v/v), 1,1-Diphenyl-2-picrylhydrazyl (DPPH), acetic acid, 2, 4, 6-tripyridyl-s-triazine (TPTZ), sodium acetate (C_2_H_3_NaO_2_·3H_2_O), sodium nitrite (NaNO_2_), Folin–Ciocalteu reagent, sodium hydroxide (NaOH) and quercetin, ascorbic acid, hydrochloric acid (HCl), ferrous sulfate (FeSO_4_·7H_2_O), ferric chloride (FeCl_3_·6H_2_O), sodium carbonate, gallic acid, and aluminum trichloride (AlCl_3_).

All reagents were provided by SC CIUPERCARIA SRL, Aghireșu-Fabrici, Cluj County Horticultural Products Laboratory, and they all were of reagent-grade purity.

### 4.4. Samples Preparation

Sampling was done for each experimental variant, formed from whole fruiting bodies of different sizes. These were washed with distilled water, cut into pieces, and frozen at −80 °C, then dried by lyophilization. The samples thus obtained were ground until a fine powder was obtained, which was kept in tightly sealed plastic bags at −20 °C until the analysis was performed.

### 4.5. Sample Extraction

For the extractions, the Pegg method [67] was used with some modifications. Thus, for each sample, 100 mL of ethanol and 10 g of powder were used in an Erlenmeyer flask covered with aluminum foil and stirred at 210 rpm for 80 min at 25 °C. After which, the extract was filtered and the residue was filtered. It was extracted two more times, then the extracts were mixed and the residual solvent was removed from the extracts in a rotary evaporator under reduced pressure at 40 °C until drying. The aqueous extract was lyophilized. The dried extracts obtained were used to make the determinations.

### 4.6. Methods Used for Analyses

The principles and procedures for selected vitamin analysis are described by Pegg and Eitenmiller [67].

### 4.7. Statistical Analysis

The processing of the obtained results was made by analyzing the polyfactorial variance, on each analyzed character, and the statistical interpretation was made with the STATISTICA 10 program with the Duncan test.

## 5. Conclusions

The nutritional value of the *Agaricus blazei* Murrill mushrooms, evaluated by their chemical composition and vitamin content, highlights the product obtained on the classic compost was improved with the protein addition of corn flour. Mushrooms harvested on this substrate have the highest levels of B1, B9, B12, PP, and vitamin Cs.

The content of ergosterol as a precursor of vitamin D2, which is generally higher in mushrooms than in other crops, has higher values in the product obtained on the classic compost, with the addition of corn flour and wheat barn.

Based on the results of the research and the general conclusions formulated, the following recommendations for production can be made: For *Agaricus blazei* Murrill mushrooms culture, synthetic wheat straw (66%), poultry manure (28%), gypsum (4%), and urea (2%) can be used with very good results. Depending on the local material resources in the area, it can be used as a culture substrate in the classical compost, consisting of garbage with bedding (93%), gypsum (5%), superphosphate (1%), and ammonium sulphate (1%).

## Figures and Tables

**Table 1:** Vitamin content of *Agaricus blazei* Murrill mushrooms.

Experimental Variants	Ergosterol (mg 100 g^−1^ dm) *	C Vitamin (mg 100 g^−1^ dm) *	B_1_ Vitamin (μg 100 g^−1^ dm) *	B_2_ Vitamin (μg 100 g^−1^ dm) *	B_9_ Vitamin (μg 100 g^−1^ dm) *	B_12_ Vitamin (μg 100 g^−1^ dm) *	PP Vitamin (mg 100 g^−1^ dm) *
V1	87.33^bc^	18.50^b^	1030^c^	4983^b^	579^d^	518^h^	43.00^e^
V2	90.17^b^	19.17^ab^	1100^b^	5450^a^	598^c^	531^gh^	45.67^d^
V3	94.00^a^	21.50^a^	1151^a^	5616^a^	631^b^	558^fg^	49.67^c^
V4	82.17^d^	12.67^gf^	900^e^	3816^e^	435^g^	703^e^	29.67^h^
V5	82.67^d^	15.00^def^	940^d^	4316^d^	458^f^	728^e^	31.67^h^
V6	84.83^cd^	15.67^cde^	956^d^	4550^cd^	475^e^	761^d^	35.00^g^
V7	82.33^d^	18.00^bc^	573^g^	4800^bc^	618^b^	828^c^	50.00^bc^
V8	82.33^d^	19.17^ab^	616^f^	4983^b^	631^b^	866^b^	52.17^b^
V9	87.50^bc^	21.67^a^	635^f^	5350^a^	671^a^	906^a^	55.33^a^
V10	73.00^f^	11.00^g^	381^i^	3183^g^	283^i^	463^i^	35.67^g^
V11	74.17^ef^	16.67^ef^	395^hi^	3450^fg^	291^i^	538^gh^	38.33^f^
V12	77.00^e^	17.00^bcd^	423^h^	3533^ef^	313^h^	573^f^	40.00^f^
SD	3.34–3.85	2.38–2.74	35.68–41.15	309.23–356.65	13.77–15.88	32.40–37.37	2.38–2.75

* Values marked with different letters are significant.

## Appendix A

**Table A1:** Experimental variants of the experimental trial.

Variant	Culture Substrate	Protein Addition
V1 (C_1_ A_1_)	classical (C1)	without protein addition (A1)
V2 (C_1_ A_2_)	classical (C1)	wheat bran 3% (A2)
V3 (C_1_ A_3_)	classical (C1)	corn flour 3% (A3)
V4 (C_2_ A_1_)	synthetic (C2)	without protein addition (A1)
V5 (C_2_ A_2_)	synthetic (C2)	wheat bran 3% (A2)
V6 (C_2_ A_3_)	synthetic (C2)	corn flour 3% (A3)
V7 (C_3_ A_1_)	mixed (C3)	without protein addition (A1)
V8 (C_3_ A_2_)	mixed (C3)	wheat bran 3% (A2)
V9 (C_3_ A_3_)	mixed (C3)	corn flour 3% (A3)
V10 (C_4_ A_1_)	Reed + horse manure (C4)	without protein addition (A1)
V11 (C_4_ A_2_)	Reed + horse manure (C4)	wheat bran 3% (A2)
V12 (C_4_ A_3_)	Reed + horse manure (C4)	corn flour 3% (A3)

**Table A2:** Recipes for substrate/compost used in experimental trial [68].

Type of Compost	Components	Quantity for 1 Tone of Compost
C1—Classical	Horse manure (horse manure and wheat bedding straw 70–75%)	500 kg
	Gypsum (calcium sulphate) CaSO_4_·2H_2_O	25 kg
	Superphosphate Ca(H_2_PO_4_)_2_·H_2_O	7 kg
	Ammonium sulphate (NH_4_)_2_SO_4_	7 kg
C2—Synthetic	Wheat straw	350 kg
	Poultry litter	150 kg
	Gypsum (calcium sulphate) CaSO_4_·2H_2_O	20 kg
	Urea CH_4_N_2_O	7 kg
C3—Mixed	Horse manure (horse manure and wheat bedding straw 70–75%)	250 kg
	Poultry litter	100 kg
	Wheat straw	150 kg
	Gypsum (calcium sulphate) CaSO_4_·2H_2_O	24 kg
	Urea CH_4_N_2_O	2 kg
C4—Original	shredded reed	100 kg
	Horse manure (horse manure and wheat bedding straw 70–75%)	200 kg
	Poultry litter	150 kg
	Gypsum (calcium sulphate) CaSO_4_·2H_2_O	24 kg
	Urea CH_4_N_2_O	2 kg

## Appendix B

The stages of mushroom culture
